# A New Preservative Compound

**Published:** 1882-06

**Authors:** 


### A NEW PRESERVATIVE COMPOUND.

Professor F. S. Barff explains in the June number of the *Month* a new antiseptic compound for the preservation of food which, it is certainly not impossible, may supersede drying and canning, and indeed all the other costly and more or less unsatisfactory processes. If this discovery after a more extensive trial than it has yet had, prove as satisfactory and as inexpensive as in the experiments that Professor Barff has made, it will be one of the most valuable chemical discoveries of recent years, and more valuable to American raisers and packers of meats than every other process heretofore discovered. In spite of refrigerator-cars and steamships, and in spite of the great improvements that have been made in canning meats, one of our greatest commercial problems still is, How can we transport our beef and pork and eventually our dairy products to Europe perfectly preserved and at a reasonable price? If this can be done, it will enlarge almost indefinitely one of our already large industries and stimulate the raising and exporting of other meats than beef and pork.

The antiseptic, as Professor Barff explains it, is a compound of glycerine and boracic acid as follows: Glycerine, boracic acid, boro-glyceride and water—by weight, ninety-two parts of glycerine and sixty-two parts of boracic acid, the odd fifty-four parts being the weight of the water which is lost as steam during the heating. "Boro-glyceride" is the name he has given to the compound. Neither glycerine nor boracic acid alone completely preserves meat, but the compound is an almost tasteless, effectual and uninjurious preservative. It does not even change the color of the meat. The method of using is to steep the fresh meat in the preserving solution, dry it in the sun and pack it in casks; or it can be left in the liquid during transportation. The cost of preserving and packing is estimated, partly, of course, by guess, inasmuch as no extensive experiment has yet been made, at a penny a pound. Among the experiments that Professor Barff has made, he mentions the following: A jar of ribs of mutton was sent last August from the Falkland Islands and the meat remained sweet and tender, and was eaten both roasted and boiled; oysters kept for three months which could not be distinguished from fresh ones, others sent raw from Jamaica to England which retained a perfect flavor; turtles and pigeons from the same place with the same satisfactory results; pigeons and beef kept seven months and made into a pie, with eggs laid six months. Furthermore, when meat has been preserved with boro-glyceride, it will keep sweet and will not turn sour for weeks after it has been cooked, even if exposed to the air. If a pan he kept containing the dilute solution, meat and poultry when brought fresh from the barnyard or the market, by being immersed in it could be kept sweet during the hottest weather. Experiments have been made also with the compound in preserving cream and butter. It does not give an unpleasant taste to milk and has in all experiments thus far made proved perfectly harmless. Cream preserved with it was sent from England to Jamaica and could not be distinguished from fresh cream and a quantity of clotted cream was sent with the same satisfactory result to Zanzibar. Butter after ten weeks of preservation, was perfectly good, and nearly a hundred persons who ate it did not detect any peculiar taste and were unwilling to believe that it was not fresh.

### A DEMONSTRATION OF A NEW METHOD.

Some time ago a most interesting demonstration of a new method of preserving meat was given at York terrace, Regent's Park, England. Instead of steeping the meat in an antiseptic, the preservative compound was introduced into the veins of the living animal, and from them into every part of the body. The sheep upon which the experiment was performed was first stunned by a blow on the head given with a wooden mallet. The left jugular vein was then laid bare, a pint of blood drawn off and about two pints of the preservative, dissolved in warm water and kept at blood heat, was injected into the opened vein. The sheep was then killed in the ordinary way, the whole occupation not occupying more than four minutes. The antiseptic used in this experiment was boracic acid, which did not in the slightest degree effect the quality or flavor of the meat, while the results of later experiments show that meat so treated will keep perfectly good without the use of ice or refrigerators for five or six weeks in summer and two or three months in cold weather.—*New York World.*

